# Fungal-mediated enhancement of yield and quality in *Chrysanthemum morifolium* under integrated rotation and organic management

**DOI:** 10.3389/fmicb.2026.1916214

**Published:** 2026-09-10

**Authors:** Guimei Tang, Guolin Huang, Yuxia Zhou, Yingshu Peng, Lili Xiang, Li Zhang, Fan Zhao, Xiuhai Zhang, Yunpeng Du, Jilong Yang, Dongliang Chen, Yuanzhi Xiao, Weidong Li, Yang Liu

**Affiliations:** 1 Hunan Horticultural Research Institute, Hunan Academy of Agricultural Sciences, Changsha, China; 2 Yuelushan Laboratory, Changsha, China; 3 Hunan Provincial Key Laboratory for Germplasm Innovation and Utilization of Landscape Plants and Flowers, Changsha, China; 4 College of Horticulture, Hunan Agricultural University, Changsha, China; 5 Beijing Academy of Agriculture and Forestry Sciences, Beijing, China

**Keywords:** *Chrysanthemum morifolium*, continuous cropping obstacles, fungal interactome, fungal positive cohesion, microbial network stability, vermicompost

## Abstract

Continuous cropping obstacles (CCOs), driven by microbial dysbiosis, hinder sustainable *Chrysanthemum morifolium* production. While rotation and organic amendments are known to alleviate CCOs, their impact on microbial interactomes remains unclear. This study evaluated 10 agronomic treatments during a full cultivation cycle, ranging from monoculture (CK) to integrated rotation systems combined with bio-organic fertilizer or vermicompost (Rot_Org and Rot_Org_Ver), in a field with five-year continuous cropping history of *Chrysanthemum morifolium*. We integrated soil physicochemical analysis, enzyme profiling, and high-throughput sequencing (16S/ITS) with phytochemical assessments. Results showed integrated management improved soil nutrients and enzyme activities (e.g., sucrase, protease), reduced bacterial *α*-diversity, but enriched beneficial fungal taxa (Ascomycota, Mortierellomycota), which responded more strongly to AN/AP shifts than bacteria. Pathogen abundance declined under vermicompost-amended rotation (Rot_Org_Ver). XGBoost modeling with SHAP interpretation identified fungal positive cohesion as the paramount biological predictor of yield, which also correlated with flavonoid and phenolic acid accumulation. We conclude that assembling a stable, cohesive fungal interactome-rather than increasing species richness-is the primary mechanism coupling soil health restoration with crop productivity, providing a theoretical framework for ecological intensification in medicinal plant cultivation.

## 1 Introduction

Intensive and continuous cultivation of high-value crops commonly leads to soil degradation and declines in ecosystem function, central components of which are reductions or imbalances in the soil microbiome that culminate in continuous-cropping obstacles (CCOs) (Chen Q. et al., 2025; Chen X. et al., 2025; Li et al., 2025). *Chrysanthemum morifolium* (*C. morifolium*) is an economically and medicinally important species with high demand for flower yield and stable phytochemical profiles (Liao et al., 2024). Its flowers are widely utilized in healthcare teas and clinical pharmacy due to their rich profile of bioactive secondary metabolites, including total flavonoids, chlorogenic acids, and various phenolic acids (Chen Q. et al., 2025; Chen X. et al., 2025). However, CCOs in *C. morifolium* manifest as yield decline, degraded flower quality (notably lower flavonoids and chlorogenic acids), and increased soil-borne disease incidence (De Corato, 2020).

The ‘soil health’ concept has recently emerged as a cornerstone for sustainable agriculture, emphasizing the capacity of a soil to function as a vital living ecosystem (De Corato, 2020; Hayden et al., 2025). In intensive monoculture systems, the depletion of soil organic matter (SOM) and the imbalance of nutrient stoichiometry (N, P, K) drive shifts in the soil microbiome, often resulting in a ‘pathogen-friendly’ environment (Chaloner et al., 2020). Rotation and organic inputs (manure, bio-organic fertilizers, vermicompost) are widely promoted to improve soil organic matter (SOM), nutrient availability and crop performance in *C. morifolium* and other cropping systems (Zhang et al., 2022). Several studies identify organic fertilization can raise available N, P, and K and increase total flavonoid and other phytochemical contents while maintaining or increasing yields (Chen Q. et al., 2025; Chen X. et al., 2025; Liu et al., 2025; Xie et al., 2022). Although numerous field studies and meta-analyses document that adding organic inputs increases crop yields, the causal chain from amendment → soil physics/chemistry → microbial processes → plant nutrition and yield is multifaceted and context-dependent (Bu et al., 2026; Fritze et al., 2024; Li et al., 2025). Different organic fractions have distinct effects on microbial growth, enzyme expression and nutrient release, deciphering which fraction drives the beneficial cascade is essential for optimized amendment design.

Organic amendments increase yield through a constellation of processes that are tightly linked to soil microbial biomass, metabolism and interaction networks - not merely by serving as nutrient inputs (Torres et al., 2015; Wu et al., 2021; Xie et al., 2015). While both bacterial and fungal communities contribute to soil functioning, accumulating evidence suggests that fungi may play a disproportionately critical role in mediating the recovery of agroecosystems under continuous cropping stress. Unlike bacteria, which primarily drive rapid nutrient turnover, fungi, with their extensive mycelial networks, act as ecosystem engineers that stabilize soil aggregates, decompose recalcitrant organic matter, and form long-term biotic interactions with plants (Chen et al., 2022; Gao et al., 2019). Fungal networks, through their persistent mycelial structures and symbiotic associations, may serve as the primary link between soil management and plant fitness (Geisen et al., 2021; Jiao et al., 2020; Possinger et al., 2022). Importantly, fungal co-occurrence networks exhibit higher structural sensitivity to environmental change and are more strongly linked to plant fitness than bacterial networks in multiple cropping systems (Banerjee et al., 2016; Possinger et al., 2022). Yet, the topological shifts in fungal co-occurrence patterns and their direct contribution to the synthesis of plant phytochemicals have not been systematically quantified in *C. morifolium* systems.

In this study, we hypothesized that: (1) integrated rotation and organic management selectively recruit beneficial fungal groups by optimizing the soil micro-environment; (2) this management reconfigures the microbial interactome toward higher complexity and cohesion; and (3) fungal network stability is the core driver for the synergistic improvement of *C. morifolium* yield and bioactive quality. To test these hypotheses, a three-year field experiment was conducted with ten agronomic treatments, combining crop rotation, bio-organic fertilizer, and vermicompost inputs. We integrated soil physicochemical analysis, enzyme profiling, high-throughput sequencing (16S/ITS), and phytochemical assessment, coupled with network analysis and machine learning models, to bridge microbial community dynamics with plant performance.

## 2 Materials and methods

### 2.1 Experimental design

The field experiment was conducted from November 2023 to November 2024 at the Chrysanthemum Research Base of Hunan Academy of Horticultural Sciences, Changsha, China (28°12′N, 113°5′E). The site was selected based on its history of five consecutive years of monoculture cultivation of *Chrysanthemum morifolium* ‘Jinsihuangju’, representing a typical continuous-cropping obstacle (CCO) scenario. The soil was a typical silt loam (pH 7.73, SOM 3.07%). A randomized complete block design was employed with ten treatments, each conducted in triplicate, resulting in a total of 30 experimental plots. Each plot measured 1.2 m × 10 m (12 m^2^) and was planted at a spacing of 40 cm × 40 cm. To minimize edge effects and treatment interference, buffer zones were maintained around each plot, and only interior plants (typically 36–40 per plot) were used for destructive sampling.

**Figure 1:**
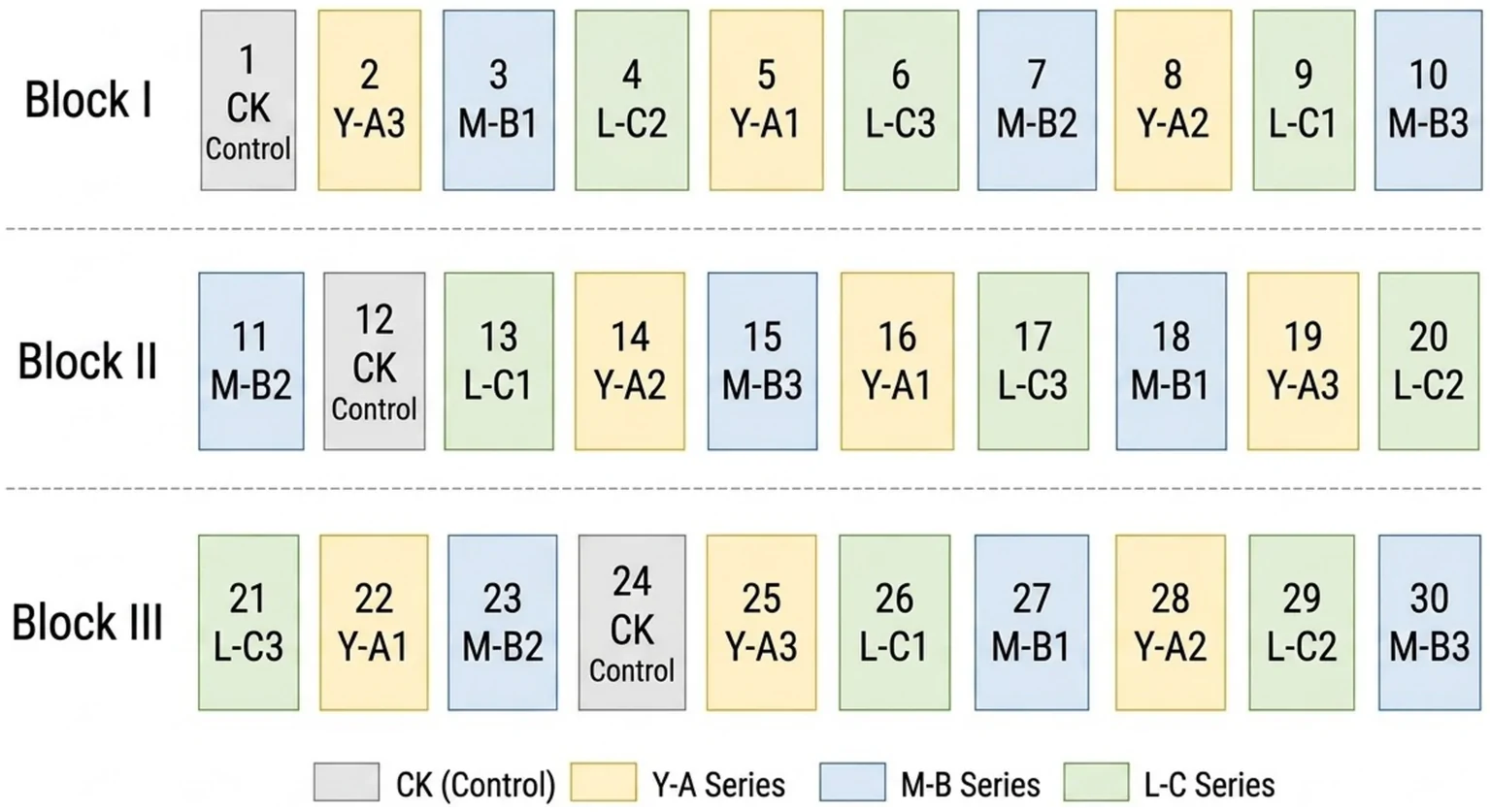
Schematic layout of experimental plots in a randomized complete block design with three replications. The experiment comprises three blocks (Block I–III), each containing one replicate of all 10 treatments, totaling 30 plots. Treatments within each block are arranged in a completely randomized order, with blocks separated by dashed lines; plot numbers 1–30 are assigned sequentially by block. Color coding indicates treatment series: gray for control (CK), yellow for Y-A series (Y-A1–Y-A3), blue for M-B series (M-B1–M-B3), and green for L-C series (L-C1–L-C3). This layout minimizes environmental heterogeneity across blocks to ensure robust statistical evaluation of treatment effects. Prefix letters (Y, M, L) indicate different rotation partner species; suffix numbers (1, 2, 3) correspond to increasing levels of organic input.

The treatments were categorized into four main regimes based on cropping history and organic supplementation: (1) continuous monoculture (CK), (2) simple crop rotation (Rot), and (3) integrated systems combining rotation with bio-organic fertilizer (Rot_Org) or vermicompost (Rot_Org_Ver) (Table 1). Treatments were randomly assigned within each block using a computer-generated randomization sequence to ensure unbiased spatial distribution and account for potential field heterogeneity (Figure 1). Crop rotation was implemented with three preceding crops: oilseed rape (*Brassica napus* L.), winter–spring radish (*Raphanus sativus* L.), and hairy vetch (*Vicia villosa*). Oilseed rape seedlings were transplanted in mid-November 2023, while radish and hairy vetch were sown during the same period. All rotation crops were terminated before flowering and incorporated or removed prior to chrysanthemum planting. ‘Jinsihuangju’ chrysanthemum was transplanted in early May 2024.

**Table 1:** Summary of experimental treatments and management regimes.

Treatment	Type	Cropping system	Soil amendment
CK	CK	Continuous *C. morifolium*	None
Y-A1	Rot	*C. morifolium* + *Brassica napus L.* rotation	None
Y-A2	Rot_Org	*C. morifolium* + *Brassica napus L.* rotation	Bio-organic fertilizer
Y-A3	Rot_Org_Ver	*C. morifolium* + *Brassica napus L.* rotation	Vermicompost
M-B1	Rot	*C. morifolium* + *Vicia villosa* rotation	None
M-B2	Rot_Org	*C. morifolium* + *Vicia villosa* rotation	Bio-organic fertilizer
M-B3	Rot_Org_Ver	*C. morifolium* + *Vicia villosa* rotation	Vermicompost
L-C1	Rot	*C. morifolium* + *Raphanus sativus L.* rotation	None
L-C2	Rot_Org	*C. morifolium* + *Raphanus sativus L.* rotation	Bio-organic fertilizer
L-C3	Rot_Org_Ver	*C. morifolium* + *Raphanus sativus L.* rotation	Vermicompost

Prior to chrysanthemum transplantation, all plots received a common base fertilizer consisting of 50 kg/667 m^2^ compound fertilizer (N: P_2_O_5_: K_2_O = 15:15:15) and 50 kg/667 m^2^ calcium magnesium phosphate or superphosphate. This basal input was uniformly applied across all ten treatments to standardize background nutrition and isolate the effects of rotation and organic amendments. The bio-organic fertilizer was produced by Wuhan Xinhongfeng Agro-Chemical Co., Ltd. (Wuhan, Hubei Province), formulated from fermented sheep manure with added *Bacillus subtilis*. The bio-organic fertilizer was applied at a rate of 0.3 kg/m^2^ (equivalent to 200 kg/667 m^2^), incorporated into soil during land preparation alongside the basal fertilizer. It contained ≥40% organic matter (OM), total nutrients (N + P_2_O_5_ + K_2_O) ≥ 5%, and had a guaranteed minimum viable count of *Bacillus subtilis* ≥2.0 × 10^8^ CFU/g. Moisture content was ≤30%. Vermicompost was also applied at 0.3 kg/m^2^ (200 kg/667 m^2^) and derived from earthworm-processed cattle manure and food waste, with an average composition of: OM = 41.6%, total N = 1.7%, P_2_O_5_ = 2.0%, K_2_O = 1.5%, C/N = 14.8, pH = 7.2, moisture = 32.1%. No mineral fertilizers or irrigation regimes were applied differentially across treatments. Drip irrigation was uniformly managed according to seasonal evapotranspiration needs. All other agronomic practices, including weeding, pest control, and harvest timing, followed local conventional protocols to minimize confounding variables (Chen Q. et al., 2025; Chen X. et al., 2025; Liao et al., 2024).

### 2.2 Soil and plant sampling

For each plot, five soil cores (0–20 cm depth) were randomly collected using a stainless-steel auger, avoiding plot edges and disturbed zones. These cores were immediately combined and thoroughly homogenized in the field to generate a single composite bulk soil sample per plot. Each plot thus constituted one biological replicate (*n* = 3 per treatment), and all downstream analyses were based on subsamples derived from this composite to prevent pseudoreplication. The composite sample was divided into two portions: one portion was stored at 4 °C for microbiological and enzymatic assays; the other was air-dried under shaded, well-ventilated conditions for physicochemical analysis. Prior to DNA extraction, fresh soil samples were further sieved (<2 mm) to remove debris while maintaining microbial community integrity.

Plant sampling was conducted at commercial maturity when ray florets were fully expanded and disc florets reached approximately 70% anthesis. Flowers were harvested in three sequential batches at 5-day intervals during morning hours to minimize diurnal variation. For yield assessment, fresh weight (FW) was recorded immediately after harvest. Subsamples of three representative flowers per plot were oven-dried at gradually increasing temperatures (from 35 °C to 55 °C) until constant weight to determine dry weight (DW) and moisture content. For phytochemical analysis, additional flower heads were rapidly freeze-dried and ground into fine powder for subsequent extraction and quantification of secondary metabolites.

### 2.3 Determination of morphological characterization, yield and quality of *Chrysanthemum morifolium*

Morphological characterization: plant height was measured as the vertical distance from the stem base to the apex, while the crown spread was determined by averaging two perpendicular projected widths. To minimize experimental error, 3–6 uniform plants per plot were tagged for consistent longitudinal measurement across growth stages. For floral morphology, inflorescence diameter was recorded at full bloom using a vernier caliper to average two perpendicular head diameters. Ray and disc florets were quantified on the same fully opened heads, employing photographic sector counting for high-density samples where necessary.

Harvest and yield quantification: flowers were harvested during morning hours when petals were horizontal and the disc floret opening reached approximately 70%. Harvesting was conducted in three successive batches at 5-day intervals. The total number of flowers per plant was recorded, and samples were immediately sealed in bags to determine fresh weight (FW) using an electronic balance. Subsequently, representative subsamples (e.g., 3 flowers per plant) were weighed individually to obtain single-flower FW. These samples were then oven-dried to a constant mass using a staged temperature regime (increasing from 35 °C to 55 °C) to calculate dry weight (DW) and moisture content.

### 2.4 Soil physicochemical analyses

Soil pH and oxidation–reduction potential (ORP) were measured in a 1:2.5 (w/v) soil-to-deionized water mixture. The suspension was shaken for 30 min and allowed to equilibrate before measurement with a digital multiparameter pH/Eh probe. Soil organic matter (SOM) was determined by the loss-on-ignition (LOI) method, where samples were dried at 105 °C for 24 h and subsequently ignited in a muffle furnace at 550 °C for 6 h.

Available nutrients were quantified according to standard protocols: available nitrogen (AN) was determined via the alkaline hydrolysis diffusion method using Conway diffusion dishes incubated at 40 °C for 24 h, followed by titration with 0.01 M HCl; available phosphorus (AP) was extracted with 0.5 M NaHCO_3_ (pH 8.5) and measured colorimetrically at 880 nm using the ascorbic acid-molybdenum blue method; and available potassium (AK) was extracted with 1.0 M NH_4_OAc (pH 7.0) and quantified by Flame Atomic Absorption Spectrometry.

### 2.5 High-throughput sequencing and bioinformatic analysis

Total microbial genomic DNA was extracted from 0.5 g of fresh bulk soil using the E. Z. N. A.® Soil DNA Kit (Omega Bio-tek, Norcross, GA, USA) following the manufacturer’s protocol. DNA integrity and concentration were verified via 1.0% agarose gel electrophoresis and a NanoDrop 2000 spectrophotometer (Thermo Scientific, USA). The V3–V4 hypervariable region of the bacterial 16S rRNA gene was amplified using the primers 338F (5’-ACTCCTACGGGAGGCAGCAG-3′) and 806R (5’-GGACTACHVGGGTWTCTAAT-3′), while the fungal ITS1 region was targeted with the primers ITS1F (5’-CTTGGTCATTTAGAGGAAGTAA-3′) and ITS2R (5’-GCTGCGTTCTTCATCGATGC-3′). PCR amplifications were conducted on a T100 Thermal Cycler (Bio-Rad, USA) under the following thermal profile: initial denaturation at 95 °C for 3 min; followed by 28 cycles of 95 °C for 30 s, 55 °C for 30 s, and 72 °C for 45 s; with a final extension at 72 °C for 5 min. The resulting amplicons were purified using a PCR Clean-Up Kit (YuHua, China), quantified via Qubit 4.0 (Thermo Fisher Scientific, USA), and pooled in equimolar concentrations. Paired-end sequencing was performed on the Illumina MiSeq PE300 platform at Biomarker Technologies Co., Ltd. (Beijing, China). Raw sequences were processed using DADA2 within the QIIME2 (v2022.2) pipeline to obtain amplicon sequence variants (ASVs). This denoising process included quality filtering, subtractions of chimeras, and dereplication, which provides higher taxonomic resolution than traditional OTU clustering. For bacterial data, trimming parameters were set as truncLen = c(280, 220); for fungal ITS, truncLen = c(250, 200); with maxN = 0 and maxEE = 2.0 per read. Merging and chimera removal followed standard DADA2 workflows. The average chimera rate across samples was 0.7% (range: 0.5–1.1%). After quality filtering, an average of 48,730 ± 3,210 high-quality non-chimeric reads per sample were retained for bacterial analysis (minimum: 45,022; maximum: 59,512). For fungal communities, an average of 65,432 ± 4,178 reads were obtained (minimum: 54,238; maximum: 69,962). All samples exceeded 20,000 usable reads and were retained for downstream analysis.

Taxonomic classification was performed using the RDP Classifier against the SILVA database (v138) for bacteria and the UNITE database for fungi. ASVs with fewer than 20 reads across all samples were discarded to ensure analytical reliability (Jiao et al., 2020). Moreover, ASVs were included in alpha- and beta-diversity analyses only if they appeared in at least two biological replicates within a treatment group or had a relative abundance > 0.01% in any sample. To ensure comparability, all diversity analyses were conducted on rarefied datasets, using a sampling depth of 45,000 reads per sample for bacteria and 50,000 reads per sample for fungi. The functional profiles were predicted using Tax4Fun2 and BugBase for bacterial communities and the FungalTraits database for fungal ecological guilds. Functional redundancy index (FRI) was calculated to evaluate the ecological resilience and functional stability of the soil bacterial community under different management regimes (Wemheuer et al., 2020). All raw sequencing data have been deposited in the National Genomics Data Center (NGDC), China National Center for Bioinformation, under BioProject accession number PRJCA069066.^1^ The submission includes demultiplexed forward and reverse reads for both 16S rRNA and ITS amplicon libraries, along with complete metadata linking each sample to its treatment, block, and sampling date.

### 2.6 Soil enzyme activities assays

Soil enzyme activities were determined using fresh soil samples (sieved to < 2 mm and stored at 4 °C) following established colorimetric protocols (Wang et al., 2022). Sucrase (Suc) activity was assayed by incubating soil with a sucrose substrate, with the released reducing sugars quantified via the 3,5-dinitrosalicylic acid (DNS) colorimetric reaction. Protease (Prot) activity was measured by incubating soil with sodium caseinate, and the released aromatic amino acids (tyrosine equivalents) were determined using Folin–Ciocalteu reagent. Alkaline phosphatase (ALP) activity was evaluated by incubating soil with *p*-nitrophenyl phosphate in a borate buffer (pH 9.4), and the liberated *p*-nitrophenol was measured colorimetrically at 400 nm. Urease (Ure) activity was determined by incubating soil with a urea solution in a citrate buffer, followed by the quantification of released NH_4_^+^ using the phenol-hypochlorite (indophenol) colorimetric method. Finally, catalase (CAT) activity was quantified via the permanganate titration method, which involved the decomposition of H_2_O_2_ by soil samples and the subsequent titration of residual H_2_O_2_ with KMnO_4_. All enzyme activities were expressed on a soil dry weight basis and measured in triplicate to ensure analytical precision.

### 2.7 Plant phytochemical analyses

Uniform *C. morifolium* flower heads were harvested at the full-bloom stage from each experimental plot. To ensure representative sampling, 20–30 flower heads were randomly collected per replicate and immediately transported to the laboratory in cooling boxes. The samples were gently rinsed with deionized water to remove surface dust and briefly blotted dry. For the analysis of heat-sensitive components (e.g., Vitamin C and soluble proteins), a portion of the fresh samples was immediately frozen in liquid nitrogen and stored at −80 °C. The remaining floral materials were subjected to a staged drying process (35 °C for 24 h, followed by 50 °C until constant weight) to preserve bioactive secondary metabolites. The dried samples were subsequently pulverized using a ball mill and passed through a 60-mesh (0.25 mm) sieve. The resulting fine powder was stored in desiccators under dark conditions prior to the extraction of flavonoids, phenolic acids, and organic acids.

The primary and secondary metabolites of *C. morifolium* were quantified using established spectrophotometric and chromatographic methods. Soluble sugars (SS) and total amino acids (AA) were determined from water-soluble extracts using the anthrone-sulfuric acid colorimetric assay and the ninhydrin colorimetric method, respectively. Soluble protein (SP) was extracted in phosphate buffer and quantified via Coomassie Brilliant Blue G-250 (Bradford) colorimetry, while Vitamin C (VC) content was measured using the 2,4-dinitrophenylhydrazine (2,4-DNPH) colorimetric assay.

Regarding secondary metabolites, total flavonoids (Flav) were extracted with ethanol under ultrasonic assistance and quantified using the aluminum-chloride colorimetric method with rutin as the standard. Individual phenolic compounds and organic acids, including 4-hydroxybenzoic acid (4-HBA), vanillin (VA), succinic acid (SA), and fumaric acid (FA), were extracted with analytical-grade solvents and quantified via High-Performance Liquid Chromatography (HPLC) or Liquid Chromatography-Mass Spectrometry (LC–MS) by comparison with authentic standards. Crude fat (CF) was determined by standard Soxhlet extraction using petroleum ether as the solvent. All spectrophotometric measurements were performed at their respective characteristic wavelengths using a UV–Vis spectrophotometer, with concentrations calculated based on standard curves and expressed on a dry weight basis.

### 2.8 Statistical analysis

Data preprocessing and basic descriptive statistics were initially performed in Microsoft Excel. To quantify the relative impact of different management regimes, the effect size of each treatment was calculated as the log-transformed ratio relative to the control (CK) using the following formula (Liu et al., 2023):

Effect size=ln(T/CK),

where T and CK represent the mean values of the treatment and control groups, respectively.

To synthesize multi-dimensional plant traits into a unified index, data were normalized using the range-scaling (min-max) function. Advanced statistical analyses were conducted in R (v4.3.0). All statistical analyses were conducted at the plot level (*n* = 3 per treatment), which represents the true biological replicate in this randomized complete block design. One-way analysis of variance (ANOVA) followed by Tukey’s HSD test (*p* < 0.05) was used to evaluate significant differences among treatments. The variation in microbial community structure was visualized via Non-metric Multidimensional Scaling (NMDS) based on Bray-Curtis distances, with significance tested using PERMANOVA. Mantel tests were employed to determine the correlations between microbial community composition and soil environmental variables. Distinctive ASVs across treatments were identified and visualized using ternary plots. For plant productivity assessment, Principal Component Analysis (PCA) was applied to yield-related traits. Spearman’s rank correlation analysis was performed to evaluate associations between phytochemical traits and soil–microbial properties. To account for multiple comparisons arising from pairwise testing across variables, *p*-values were adjusted using the Benjamini-Hochberg procedure to control the false discovery rate (FDR). Correlations with an adjusted *q* < 0.05 were considered statistically significant. All analyses were conducted in R (v4.3.0) using the ‘corrplot’ and ‘p.adjust’ functions.

Microbial co-occurrence networks were constructed separately for three key management regimes: simple rotation (Rot), rotation with bio-organic fertilizer (Rot_Org), and rotation with vermicompost (Rot_Org_Ver). Each network was inferred from 9 independent biological samples (3 crop rotation types × 3 replicates), ensuring sufficient replication for correlation analysis. Only abundant ASVs (relative abundance > 0.1% across all samples within a treatment group) were included to reduce noise from rare taxa. Pairwise Spearman’s rank correlations were calculated within each regime, and a robust edge was defined as |*r*| > 0.8 and *p* < 0.01. It enables the identification of strong, consistent associations under standardized agronomic conditions (Layeghifard et al., 2017; Jiao et al., 2022). The resulting network properties, including the number of nodes, edges, average degree, clustering coefficient, and betweenness centrality, were calculated to characterize topological structure. All network visualizations and topological calculations were performed using the igraph and Hmisc packages in R.

The calculation of positive and negative cohesion indices, as well as network stability, followed the framework described by Herren and McMahon (2017). For each species in the network, its positive connectivity was computed as the average of all significant positive correlations with other taxa, while its negative connectivity was calculated as the average of all significant negative correlations. The overall positive cohesion of the network was then determined as the abundance-weighted mean of all individual species’ positive connectivities, reflecting the strength of cooperative interactions within the community. Similarly, negative cohesion was calculated as the abundance-weighted mean of negative connectivities, representing the extent of competitive or antagonistic associations. To evaluate network stability, a Stability Index was derived by taking the ratio of positive cohesion to the absolute value of negative cohesion. A Stability Index greater than 1 indicates that positive interactions dominate the network, typically associated with higher structural resilience and functional stability, whereas a value less than 1 suggests a system more prone to instability due to prevalent negative interactions (Hernandez et al., 2021).

To identify the key biological drivers of *C. morifolium* yield, an extreme gradient boosting (XGBoost) regression model was implemented in Python (v3.9) using the xgboost and scikit-learn libraries. Given the limited sample size, a 60%/40% train-test split (n_train = 18, n_test = 12) was applied using stratified random sampling to ensure representative distribution of yield values in both sets. Hyperparameter tuning was performed via 5-fold cross-validation within the training set using grid search. Final parameters: learning_rate = 0.1, max_depth = 3, n_estimators = 100, subsample = 0.8, colsample_bytree = 0.8. Model performance was evaluated on the independent test set. The model’s interpretability was enhanced through SHAP (SHapley Additive exPlanations) value analysis, which quantified the contribution and global importance of each soil and microbial predictor to the final crop yield. The final model achieved an *R*^2^ = 0.51 and RMSE = 0.48 (MAE = 0.36) on the held-out test set. While this indicates moderate predictive accuracy, it demonstrates that the model captures meaningful patterns beyond random chance. Repeated runs with different random seeds yielded consistent SHAP rankings, supporting the stability of variable importance estimates.

## 3 Results

### 3.1 Integrated rotation and organic management significantly enhanced soil nutrient availability and C-N cycling enzyme activities

**Figure 2:**
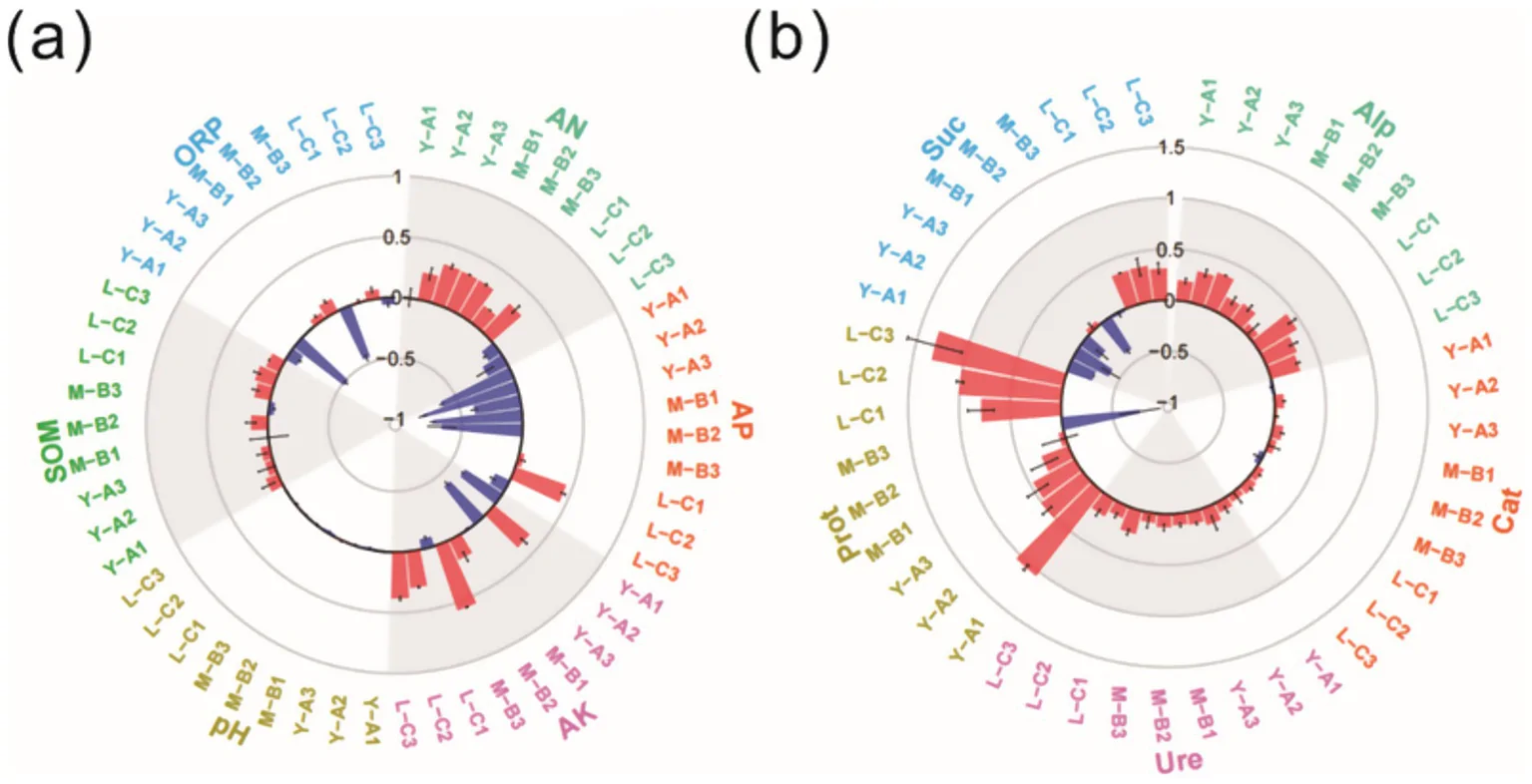
Relative effect sizes of soil physicochemical properties **(a)** and enzyme activities **(b)** compared to the control (CK). Circular bar plots illustrate the effect sizes of different treatments relative to the control (CK). Bar length and direction indicate the magnitude and direction of the effect size, with positive and negative values representing increases or decreases relative to the reference condition. Y-series: *C. morifolium*–*Brassica napus* rotation; M-series: –*Vicia villosa*; L-series: –*Raphanus sativus*. A1/B1/C1: Rot; A2/B2/C2: Rot_Org; A3/B3/C3: Rot_Org_Ver.

The integrated rotation and organic management significantly altered soil nutrient availability compared to the continuous monoculture (CK) (Supplementary Table S1). Among the ten treatments, the L-C2 (*C. morifolium* + *Raphanus sativus L.* rotation) and L-C3 (*C. morifolium* + *Raphanus sativus L.* rotation + Vermicompost) regimes emerged as the most effective in enhancing soil fertility. Specifically, L-C2 exhibited a comprehensive improvement in available nutrients, with available nitrogen (AN), phosphorus (AP), and potassium (AK) levels significantly exceeding those of the CK (*p* < 0.05). In contrast, while M-B3 (*C. morifolium* + *Vicia villosa* rotation + Vermicompost) showed relatively lower AN and AP levels, it recorded the highest AK concentration (33.67 mg/kg). To further quantify the magnitude of management-induced changes, the log-transformed effect sizes of soil parameters relative to the CK were visualized using circular heatmaps (Figure 2). Regarding soil chemistry (Figure 2a), a predominant positive effect (indicated by red bars) was observed across most treatments for available nitrogen (AN), phosphorus (AP), and potassium (AK), demonstrating a substantial enhancement in nutrient supply post-intervention. Soil organic matter (SOM) exhibited a consistent upward trend across all managed groups, reinforcing the efficacy of integrated rotation and organic amendments in carbon sequestration. Conversely, soil pH displayed negative effect sizes (blue bars) in several treatments, particularly within the M (*C. morifolium* + *Vicia villosa*) and L (*C. morifolium* + *Raphanus sativus L.*) series, indicating a subtle yet detectable acidification of the soil matrix relative to the monoculture control.

A parallel enhancement was observed in soil enzyme activities (Figure 2b). The treatments generally exerted a stimulatory effect on Sucrase (Suc), Alkaline Phosphatase (Alp), Catalase (Cat), Urease (Ure), and Protease (Prot). Among these, Suc and Prot exhibited the most pronounced positive effect sizes, with the highest magnitudes recorded in the L-C and M-B subgroups. This marked response underscores a significant intensification of carbon and nitrogen cycling potentials under these specific integrated regimes, aligning with the observed increases in nutrient availability.

### 3.2 Organic management reduced bacterial diversity but strongly promoted fungal richness and evenness

**Figure 3:**
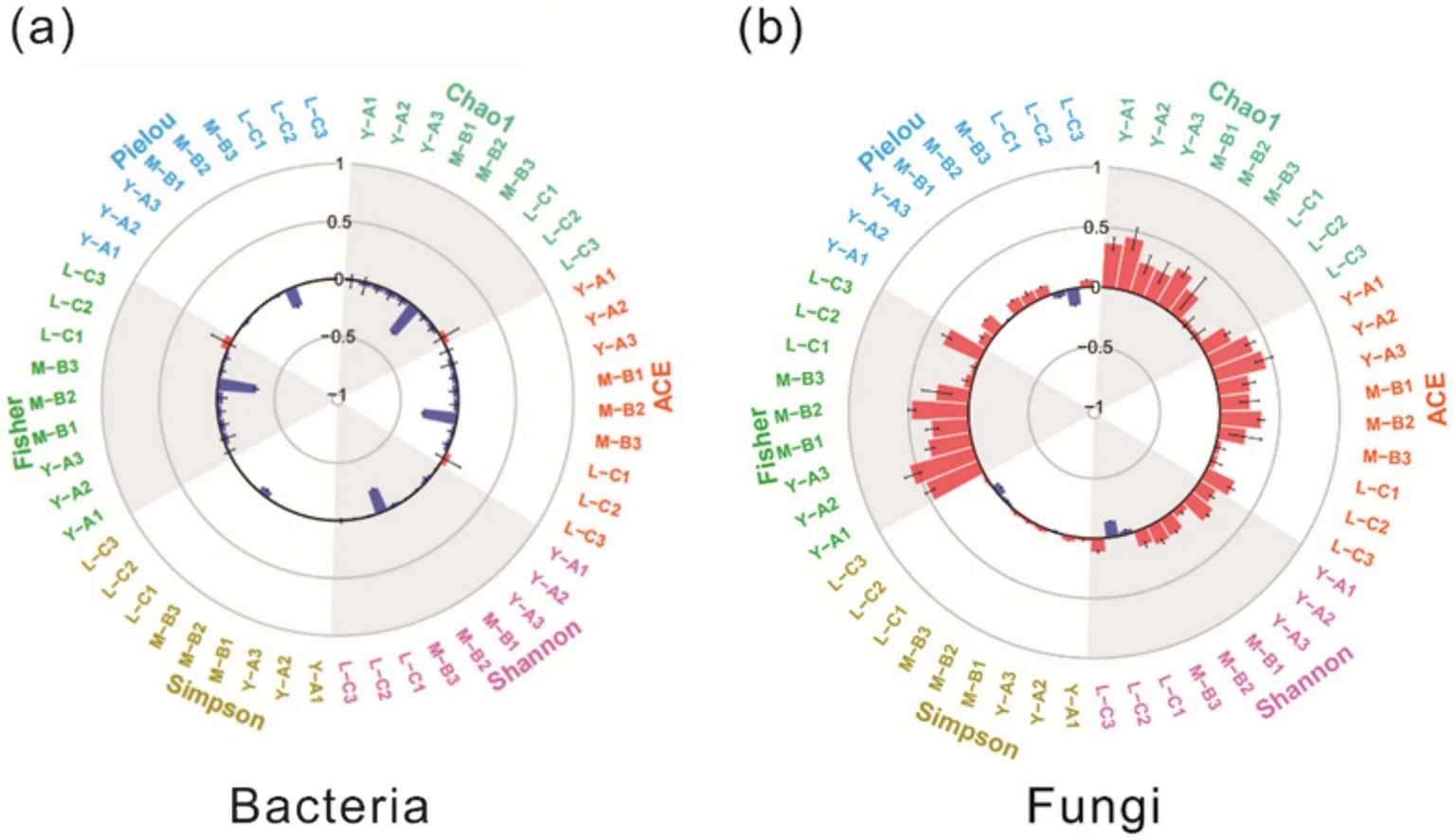
Relative effect sizes of microbial *α*-diversity indices compared to the control (CK) for bacterial **(a)** and fungal **(b)** communities. Y-series: *C. morifolium*–*Brassica napus* rotation; M-series: –*Vicia villosa*; L-series: –*Raphanus sativus*. A1/B1/C1: Rot; A2/B2/C2: Rot_Org; A3/B3/C3: Rot_Org_Ver.

The response of soil microbial *α*-diversity to the experimental treatments exhibited a marked taxonomic divergence (Figure 3). Regarding bacterial diversity (Figure 3a), the effect sizes for species richness (Chao1 and ACE) and diversity indices (Shannon and Simpson) were predominantly negative or neutral across the majority of management regimes. This trend indicates that the transition from continuous monoculture to integrated rotation and organic management generally led to a contraction in bacterial α-diversity relative to CK. Conversely, fungal communities displayed a robust and positive response to nearly all treatments (Figure 3b). Strong positive effect sizes were observed for all fungal diversity metrics, with the most substantial increases recorded in the Chao1 and ACE indices. These results suggest that the integration of crop rotation with organic amendments significantly stimulated fungal proliferation and species richness.

### 3.3 Fungal community assembly is more sensitive than bacterial to changes in available nitrogen and phosphorus

**Figure 4:**
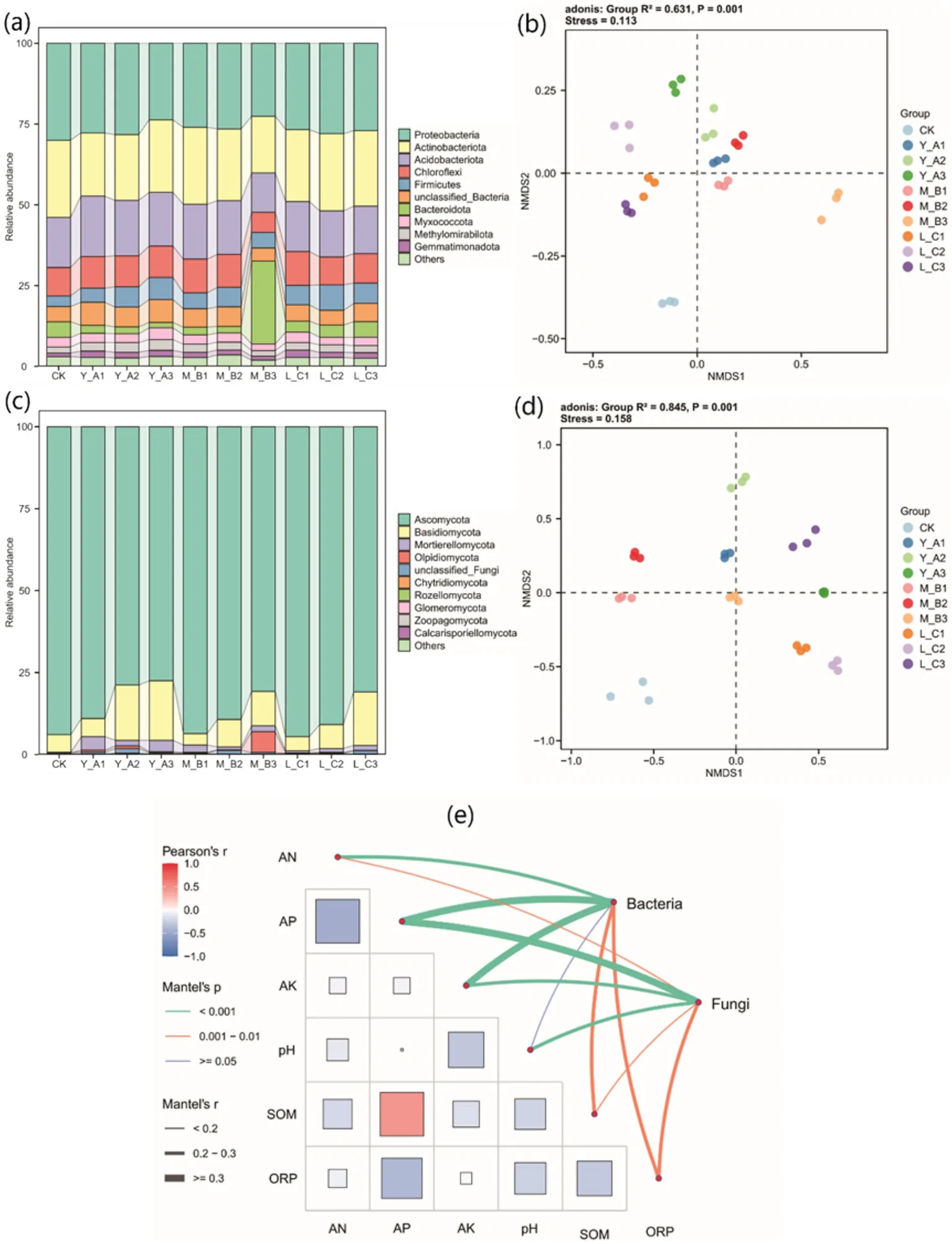
Microbial community composition, *β*-diversity, and associations with soil factors **(a,c)**. Relative abundance of bacterial and fungal communities at the phylum level across treatments, compared to CK **(b,d)**. Non-metric multidimensional scaling (NMDS) ordinations based on Bray–Curtis dissimilarity showing differences in bacterial and fungal community structures among treatments. **(e)** Correlations between soil properties and microbial community composition. Square size and color indicate Pearson’s correlation coefficients, while connecting lines represent Mantel test results, with line thickness and color corresponding to Mantel’s *r* and significance level. Y-series: *C. morifolium*–*Brassica napus* rotation; M-series: –*Vicia villosa*; L-series: –*Raphanus sativus*. A1/B1/C1: Rot; A2/B2/C2: Rot_Org; A3/B3/C3: Rot_Org_Ver.

The taxonomic composition and structural shifts of soil microbiota were deeply influenced by the management regimes (Figure 4 and Supplementary Figures S1, S2). *Proteobacteria*, *Actinobacteriota*, and *Acidobacteriota* constituted the dominant bacterial phyla across all samples (Figure 4a). The ternary plots (Supplementary Figure S1a) illustrate the distribution and enrichment of microbial ASVs across different treatment combinations. A substantial number of ASVs were significantly enriched in the rotation and organic amendment groups. These enriched bacterial ASVs were primarily affiliated with *Proteobacteria* and *Actinobacteriota*, which were positioned towards the vertices representing integrated treatments. This spatial distribution suggests that organic-enriched environments provide favorable niches for these fast-growing, nutrient-cycling copiotrophs.

The fungal communities were characterized by a heavy dominance of Ascomycota; however, a notable expansion of *Mortierellomycota* was observed in specific rotation groups, particularly M-B3 and L-C1 (Figure 4c), suggesting that diversified cropping systems create niche spaces for specific fungal lineages. The ternary plots (Supplementary Figure S1b) exhibited a more pronounced enrichment pattern. Many enriched ASVs belonged to the Ascomycota and Mortierellomycota phyla. Notably, the high density of enriched points towards the Rot_Org and Rot_Org_Ver vertices underscores the superior capacity of radish-based rotation combined with organic/vermicompost to foster a diverse and robust fungal assemblage.

Non-metric multidimensional scaling (NMDS) analysis revealed that management types were the primary determinants of community assembly (Figures 4b,d). Bacterial communities exhibited a distinct separation between the CK and organic-enriched treatments (Rot_Org and Rot_Org_Ver), with the grouping effect explaining a significant proportion of the variance (*R*^2^ = 0.631, *p* = 0.001). Interestingly, the fungal communities exhibited even tighter clustering and a higher degree of structural sensitivity to treatment types (*R*^2^ = 0.845, *p* = 0.001). This higher *R*^2^ value suggests that fungi are more responsive ‘sentinels’ of agronomic intervention than bacteria in this continuous cropping system.

Mantel tests were performed to bridge the gap between soil properties and microbial succession (Figure 4e). The results demonstrated that AN, AP, and SOM were the most influential environmental drivers, exhibiting highly significant correlations (*p* < 0.001) with both bacterial and fungal community structures. These findings indicate that the optimization of soil nutrient status through rotation and organic amendments is the fundamental mechanism driving the reconfiguration of the soil microbiome.

### 3.4 Integrated management suppressed plant pathogens and altered microbial metabolic strategies toward anaerobiosis

**Figure 5:**
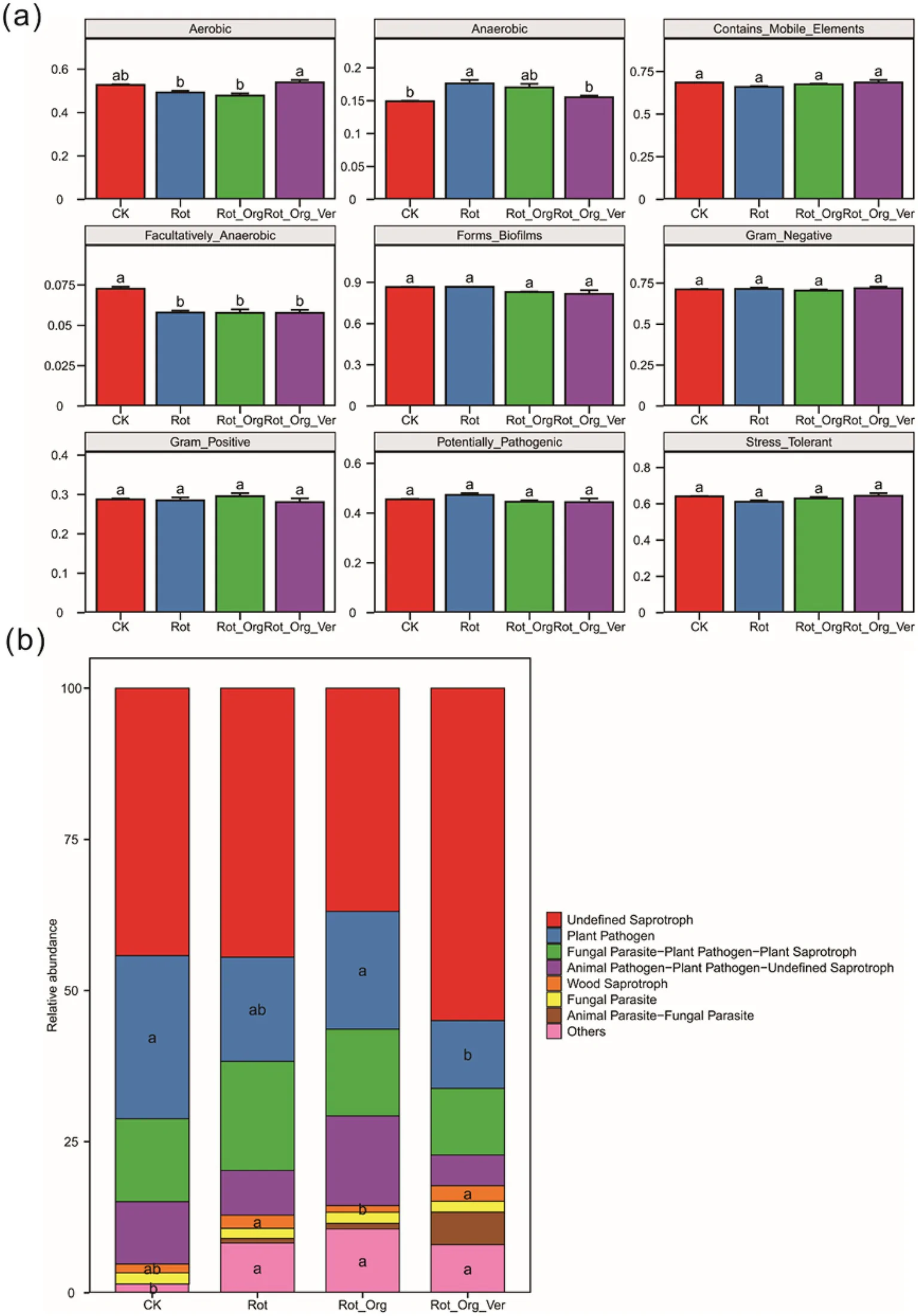
Predicted bacterial functional traits based on BugBase database **(a)** and fungal trophic modes based on FungalTraits database **(b)**.

The functional potential and ecological guilds of the soil microbiome were significantly modulated by the integrated management regimes (Figure 5). The Bacterial Functional Redundancy Index (FRI) was analyzed to assess the potential resilience of the soil microbial ecosystem across the three management regimes (Supplementary Figure S2). Predicted functional profiles of the bacterial community, generated via Tax4Fun2, suggested an enrichment of lineages associated with anaerobic metabolism (Figure 5a). A higher inferred abundance of such taxa was observed in Rot and Rot_Org treatments compared to CK (*p* < 0.05). Conversely, facultatively anaerobic bacteria exhibited a consistent decline in all rotation-based treatments relative to the continuous monoculture. These phenotypic transitions suggest that rotation and organic fertilization may alter soil micro-environments (e.g., through changes in porosity or oxygen consumption during organic matter decomposition), favoring specific metabolic strategies. As Supplementary Figure S2 showed, the lowest Functional Redundancy Index (FRI) was observed in the Rot_Org_Ver treatment, indicating reduced overlap in metabolic functions among bacterial taxa. This suggests a shift toward a less redundant, potentially more functionally specialized community. While such a configuration may imply diminished functional buffering capacity under disturbance, it may also reflect higher resource-use efficiency under stable, nutrient-rich conditions.

The fungal functional landscape was tentatively assigned using the FungalTraits database, indicating a predominance of undefined saprotrophic guilds. Notably, the predicted abundance of pathogen-associated taxa was reduced, especially in Rot_Org_Ver, compared to CK (*p* < 0.05) (Figure 5b), which is consistent with a shift toward a more suppressive microbiome composition. This reduction in potential soil-borne pathogens suggests that the combination of crop rotation and complex organic amendments fosters a disease-suppressive environment, effectively mitigating the biological constraints associated with continuous cropping obstacles.

### 3.5 Organic amendments increased fungal network connectivity, while bacterial networks became more fragile

**Figure 6:**
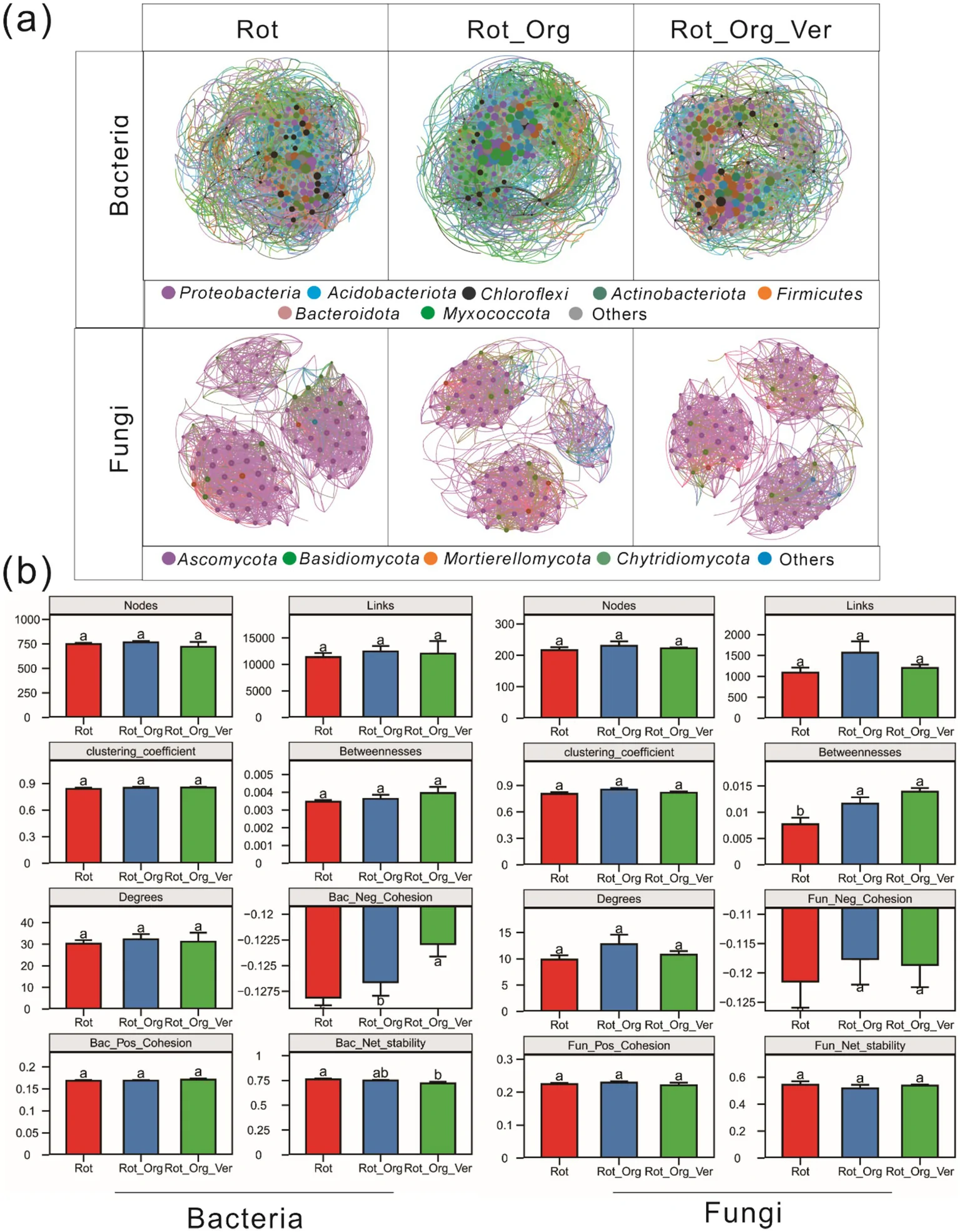
Microbial co-occurrence networks **(a)** and network topological properties **(b)** among different treatments. Nodes represent taxa (colored by phylum), and edges represent significant correlations. Different letters indicate significant differences among treatments.

To elucidate the inter-taxa interactions and community stability under different management regimes, co-occurrence networks were constructed for both bacterial and fungal communities (Figure 6). Visual inspection of the co-occurrence networks indicated that the addition of organic matter (Bio-organic and Vermicompost) increased the complexity of bacterial interactions, characterized by a higher density of nodes and edges in the Rot_Org and Rot_Org_Ver treatments. However, the network stability (calculated via robustness/cohesion metrics) showed an inverse trend; it remained highest in the CK and simple Rot groups but significantly decreased in the Rot_Org_Ver group (*p* < 0.05). This suggests that while organic enrichment promotes bacterial interaction frequency, it may simultaneously lead to a more fragile network structure that is highly sensitive to external resource inputs.

The fungal networks exhibited a distinct response to organic amendments, particularly in terms of node importance. The betweenness centrality-a key metric reflecting the influence of ‘bridge’ taxa within the network-was significantly higher in the Rot_Org and Rot_Org_Ver groups compared to the simple Rot treatment (*p* < 0.05). This increase suggests that organic amendments recruit and empower key fungal lineages that act as essential connectors, facilitating information or resource flow across the fungal interactome.

### 3.6 Crop yield was best predicted by fungal positive cohesion, not soil nutrients or bacterial metrics

**Figure 7:**
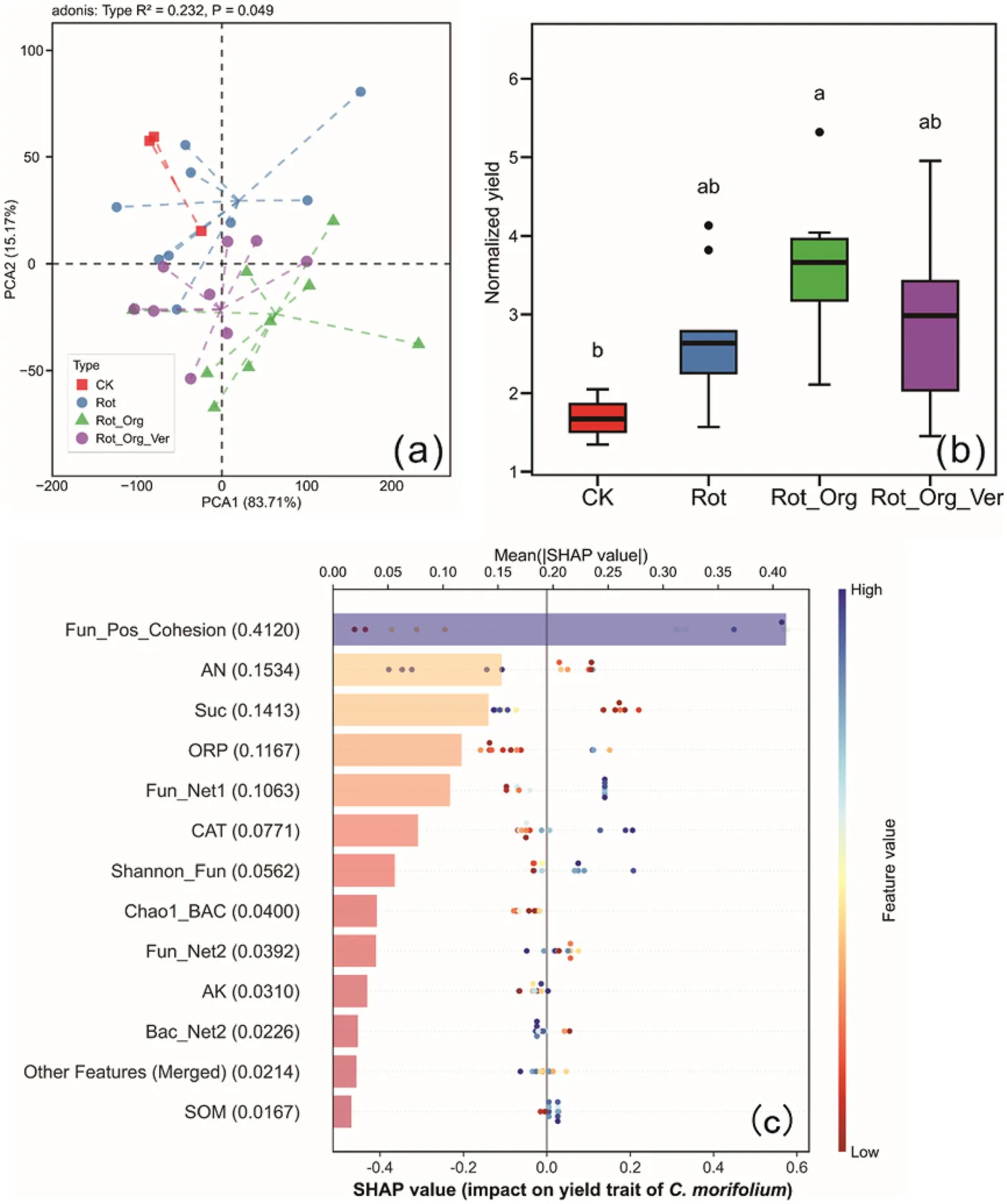
Yield variation based on principal component analysis (PCA) **(a)**, normalized yield of *C. morifolium* under different treatments **(b)**, and SHAP summary plot showing the relative importance and direction of key predictors in the yield prediction model **(c)**. Bars represent mean absolute SHAP values, while colored points show SHAP values for individual samples, with colors indicating feature values.

The integrated management regimes led to a substantial enhancement in *C. morifolium* productivity (Figure 7). One-way ANOVA revealed that fertilization significantly influenced several key growth and morphological parameters (Supplementary Table S2), while inflorescence diameter and fresh weight per plant remained stable across groups without significant differences (*p* > 0.05) likely due to genetic or environmental constraints, the various fertilization regimes significantly modulated plant morphology and yield structure, with the Y-A2 (Rot_Org) treatment achieving the highest yield among all treatments. This treatment not only significantly enhanced vegetative growth parameters such as plant height (55.83 cm) and crown spread but also exhibited superior performance in key reproductive outputs; specifically, the number of flowers per plant (87.00) and the ray floret count (365.33) were both significantly higher than those of the control.

Based on the normalized yield data (Figures 7a,b), all experimental treatments significantly outperfomed the continuous monoculture control (CK). The Rot_Org (Rotation + Bio-organic fertilizer) treatment achieved the highest median yield, which was significantly greater than that of the CK (*p* < 0.05). These results confirm that the transition from monoculture to diversified rotation combined with organic amendments effectively alleviates the ‘yield gap’ typically associated with continuous cropping obstacles.

To decipher the complex biological and physicochemical drivers of *C. morifolium* productivity, an XGBoost regression model coupled with SHAP (SHapley Additive exPlanations) value analysis was implemented (Figure 7c). The SHAP summary plot identified fungal positive cohesion (Fun_Pos_Cohesion) as the most influential feature driving *C. morifolium* yield, exhibiting the highest global importance score. This finding underscores that the internal connectivity and cooperative interactions within the fungal network are more critical for yield than individual taxa abundance (Supplementary Figure S3). Other critical contributors to the yield model included soil available nitrogen (AN), sucrase activity (Suc), and oxidation–reduction potential (ORP). The high ranking of these variables underlines a coupled mechanism: where the intensification of fungal network interactions, the optimization of nutrient availability, and the stabilization of soil redox status collectively determine the final crop productivity.

### 3.7 Enhanced flavonoid and phenolic acid accumulation correlated strongly with fungal network stability and sucrase activity

**Figure 8:**
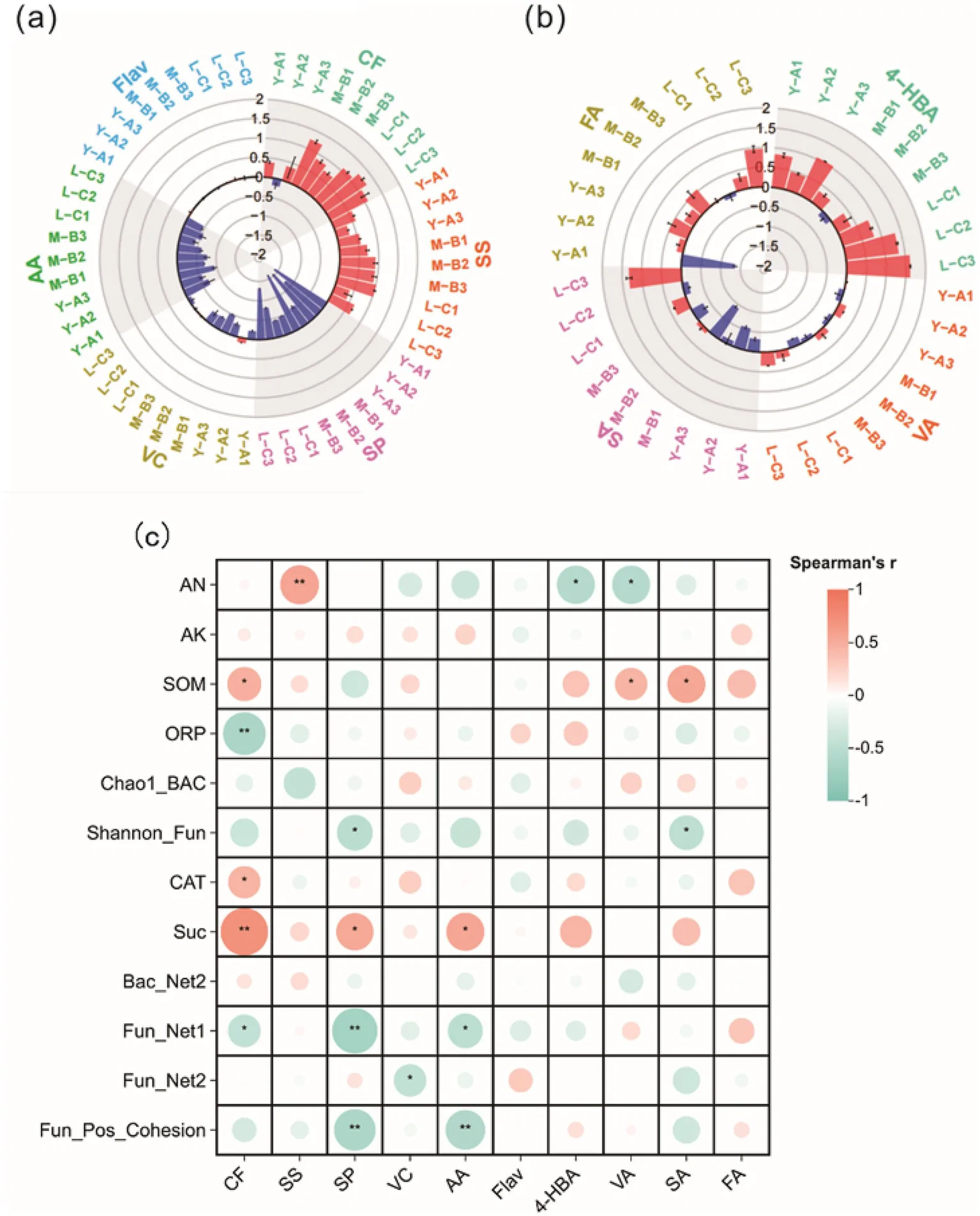
Relative effect sizes of plant nutrients **(a)** and secondary metabolites **(b)** compared to the control (CK) and their correlations with soil-microbiome features **(c)**. Circle color indicates correlation direction (positive or negative), and circle size reflects correlation strength. Asterisks denote significance levels (*p < 0.05; **p < 0.01).

The nutritional and medicinal quality of *C. morifolium* was significantly enhanced by the integrated management regimes, as evidenced by the shifts in plant nutrients and secondary metabolites (Figure 8). Analysis of the effect sizes revealed that nearly all measured phytochemicals were positively impacted by the treatments relative to the CK (Figure 8a). Notably, Total Flavonoids (Flav) and Vitamin C (VC) exhibited substantial increases, which are critical for the antioxidant properties of the flower. Furthermore, key phenolic acids and organic acids, including fumaric acid (FA), succinic acid (SA), and vanillin (VA), showed significant accumulation across the rotation and organic-amended groups. These results demonstrate that optimized soil management not only boosts yield but also intensifies the biosynthesis of health-promoting secondary metabolites.

To decipher the drivers of nutrient quality, Spearman correlation analysis was performed between phytochemical contents and soil-microbial properties (Figure 8c). Soil available nitrogen (AN) and sucrase activity (Suc) were identified as the primary chemical drivers, showing significant positive correlations with a wide array of quality indicators, including crude fat (CF), soluble protein (SP), and amino acids (AA). Importantly, fungal positive cohesion (Fun_Pos_Cohesion)-the key network feature previously linked to yield-also exhibited strong positive correlations with several essential metabolites. This indicates that a highly cooperative and stable fungal network does not only determine the quantity of the harvest but also plays a fundamental role in regulating the metabolic profile and overall quality of the *C. morifolium*.

To further elucidate the potential biological pathways linking microbial taxa to plant metabolic performance, we constructed a comprehensive association network integrating dominant bacterial and fungal genera, soil enzyme activities, and key phytochemicals (Supplementary Figure S4). This visualization reveals distinct clusters of beneficial microbes co-varying with specific enzymatic functions and plant metabolites. Notably, genera such as *Bacillus*, *Scedosporium*, and *Didymella* show strong positive associations with sucrase and protease activities, which in turn correlate with increased levels of soluble sugars, amino acids, and flavonoids. Additionally, *Cystobasidium* and *Mortierella* are linked to ALP and catalase activities, aligning with improved phosphorus availability and antioxidant capacity in plants.

## 4 Discussion

### 4.1 Improved soil fertility under integrated management is driven by sustained release of nutrients from organic inputs

The long-term continuous monoculture of *C. morifolium* typically leads to ‘soil sickness’, characterized by nutrient depletion and the accumulation of autotoxic compounds (Li et al., 2023; Guo et al., 2024). Our results demonstrate that integrated management-specifically organic-based rotation (L-C2) and vermicompost supplementation (L-C3)-effectively reverses these trends by significantly elevating the bioavailability of essential nutrients (Supplementary Table S1). The observed increase in available nitrogen (AN), phosphorus (AP), and potassium (AK) aligns with the broader consensus that organic amendments improve microbially-mediated soil functioning in agroecosystems. Unlike mineral fertilizers, which provide immediate but transient nutrient pulses, bio-organic fertilizers and vermicompost foster a slow-release reservoir of organic matter (SOM), which acts as a fundamental driver for soil fertility restoration (Li et al., 2023; Xie et al., 2022).

The intensification of C and N cycling potentials, evidenced by the marked increase in sucrase (Suc) and protease (Prot) activities in the L-C and M-B subgroups (Figure 2b), suggests a functional revitalization of the soil matrix. Soil enzymes serve as sensitive indicators of management-induced changes; their stimulation indicates that the transition from monoculture to integrated systems alleviates the biochemical constraints associated with continuous cropping (Guo et al., 2024; Zhang et al., 2022). This is particularly evident in the radish-rotation (Rot_Org) treatments, where the combined effect of diversified root exudates and organic inputs likely optimized the metabolic niches for nutrient-mineralizing microbes. Furthermore, while the subtle acidification (decline in pH) observed in the rotation series might be attributed to the secretion of organic acids during residue decomposition or enhanced microbial respiration, it may also facilitate the mobilization of fixed phosphorus and micronutrients, thereby contributing to the overall enhancement of nutrient bioavailability (Liu et al., 2020; Perillo et al., 2019). These findings suggest that the optimization of soil nutrient status through organic supplementation is not merely a quantitative increase in inputs but a qualitative reconfiguration of the soil’s biogeochemical environment, which is essential for sustaining high-yield *C. morifolium* production.

Similarly, while the moderate decrease in soil pH observed in rotation series (e.g., M-B and L-C groups) likely facilitates phosphorus solubilization through organic acid release and proton-driven desorption (Liu et al., 2020), chronic acidification poses non-negligible risks. In silt-loam soils like ours, continued pH decline below 6.5 could eventually lead to aluminum (Al^3+^) or manganese (Mn^2+^) solubilization, inducing root toxicity and impairing nutrient uptake (Kochian et al., 2004; Neina, 2019). Although current pH levels (lowest: ~7.1) remain well above the threshold for such toxicities, multi-year extension of these practices warrants careful monitoring.

### 4.2 Bacteria and fungi respond differently to management: bacteria decline in diversity, while fungi form stable cooperative networks

The experimental treatments induced a profound taxonomic divergence in microbial successional trajectories. The contraction of bacterial *α*-diversity alongside the robust expansion of fungal richness (Chao1 and ACE indices) (Figure 3b) highlights the differential sensitivity of these two domains to agronomic intervention. This disparate response reflects the ‘copiotrophic hypothesis’ (Stone et al., 2023; Wang et al., 2021), where nutrient-rich organic amendments favor specific fast-growing bacterial phyla, such as Proteobacteria and Actinobacteriota, potentially leading to the competitive exclusion of rare taxa and a subsequent decline in overall bacterial diversity (Malard et al., 2022; Turner et al., 2019). This aligns with prior studies showing that bacterial and fungal communities exhibit distinct sensitivities to changes in fertilization and rotation regimes (Malard et al., 2022). By contrast, fungal communities more readily exploit complex, recalcitrant organic substrates and form diverse hyphal networks that capitalize on the heterogeneous carbon architecture provided by increased SOM and diverse rotation residues (Klawonn et al., 2021), consequently, saprotrophic and mycelial taxa (notably many Ascomycota and Mortierellomycota lineages) can proliferate and diversify when organic inputs and cropping diversity create new niche space, which explains the strong positive effect sizes across fungal richness and abundance metrics in our managed plots (Figure 2b).

Fungal communities, however, emerged as more responsive ‘sentinels’ of management shifts, as indicated by the higher *R*^2^ value (0.845) in the NMDS analysis compared to bacteria (Figures 4b,d). The significant expansion of *Mortierellomycota* and *Ascomycota* in integrated treatments suggests that organic-enriched environments and diversified residues create specialized niches for saprotrophic and beneficial fungal lineages. Notably, the enrichment of *Trichoderma* species has been documented in soils receiving organic-mineral combinations, providing a biological basis for enhanced ecosystem stability and pathogen suppression.

Mantel tests confirmed that SOM, AN, and AP were the primary environmental filters driving this successional reconfiguration (Figure 4c). The shift in bacterial functional traits toward anaerobic phenotypes further indicates that organic management alters soil micro-environments, likely through changes in oxygen consumption during decomposition or modifications in soil porosity (Giacometti et al., 2021; Kracmarova et al., 2022). While the shift toward fungal dominance under integrated organic management correlates with improved yield and disease suppression, such restructuring may also entail potential trade-offs for long-term agroecosystem resilience. The observed reduction in bacterial *α*-diversity and functional redundancy index (FRI) in high-input treatments, particularly Rot_Org_Ver, suggests a narrowing of metabolic capabilities within the bacterial community. Lower FRI implies diminished functional insurance, meaning fewer taxa share overlapping ecological roles, thereby increasing the risk of ecosystem function collapse under future stressors such as drought, pathogen invasion, or chemical contamination (Wemheuer et al., 2020; Louca et al., 2018).

Although fungal networks exhibited enhanced cohesion and were strongly linked to plant performance, there is currently no direct evidence that they can fully compensate for the loss of bacterial functional breadth. Indeed, bacteria remain essential for rapid nutrient cycling, nitrification, and degradation of labile compounds—functions less efficiently performed by most fungi (Jiao et al., 2020). A system increasingly reliant on fungal-mediated processes may therefore become more specialized but potentially less adaptable over time (Trivedi et al., 2020). Additionally, the significant reduction in predicted fungal pathogens, particularly under Rot_Org_Ver, highlights another pathway: disease suppression via competitive exclusion and niche occupation by beneficial saprotrophs such as *Mortierellomycota* and *Ascomycota* (Deng et al., 2026). These lineages are known to produce antifungal metabolites and promote systemic resistance in host plants (Taheri et al., 2026; Jung et al., 2024).

### 4.3 Fungal network cohesion—not taxonomic richness—is the key biological predictor of yield and quality

Based on the phytochemical data presented in Supplementary Table S3, the Y-A2 treatment is identified as the optimal regime for enhancing crop quality. The superiority of Y-A2 is demonstrated by its significant impact on the accumulation of both primary and secondary metabolites. Furthermore, Y-A2 showed a robust enrichment in key phenolic acids (e.g., vanillin and succinic acid) and essential primary metabolites like soluble proteins (SP) and amino acids (AA) compared to the control (CK-1) and other rotation series. While treatments such as L-C2 demonstrated competitive levels in certain phenolic compounds, Y-A2 provided the most balanced and comprehensive improvement across the entire phytochemical profile. These findings suggest that the specific nutrient formulation and concentration in the Y-A2 regime effectively stimulate the biosynthetic pathways of bioactive compounds, thereby maximizing the medicinal and nutritional value of *C. morifolium*.

The SHAP-based predictive modeling identified fungal positive cohesion as the most influential driver of *C. morifolium* yield, transcending the importance of traditional chemical indicators like AN or SOM (Figure 7c and Supplementary Figure S3). This underscores a shift in our understanding of productivity: from the abundance of individual taxa to the strength of cooperative interactions within the fungal interactome (Banerjee et al., 2016; Klawonn et al., 2021). The strong positive correlation between fungal positive cohesion and both yield and phytochemical accumulation suggests that stable fungal network structures may play a functional role in enhancing crop performance. While we cannot establish causation from observational data, this pattern generates several testable hypotheses: (i) cooperative fungal interactions could enhance resource translocation via mycelial networks; (ii) enriched saprotrophic fungi might improve soil aggregation through hyphal binding or glomalin-related compounds; (iii) suppression of pathogen abundance may be linked to competition or antibiosis from beneficial taxa; and (iv) systemic modulation of plant metabolism could be induced by microbial signals or improved nutrient status. These pathways might effectively mitigate the biological barriers of continuous cropping obstacles (Chen Q. et al., 2025; Chen X. et al., 2025; Yang et al., 2023).

The reduced predicted abundance of plant-pathogenic fungi in the Rot_Org_Ver group may contribute to improved yield. Integrated management appears indicative of a microbiome state that supports lower disease pressure, potentially reducing immune costs and redirecting resources toward growth and secondary metabolism (Guo et al., 2024; Yang et al., 2023). Fungal positive cohesion was positively correlated with key phytochemicals including flavonoids and phenolic acids (Figure 8c), raising the hypothesis that fungal network stability may influence plant metabolic regulation through indirect pathways such as nutrient availability or microbial signaling (Chamkhi et al., 2022; Ishfaq et al., 2023). The significant positive correlations between sucrase activity, AN, and bioactive quality indicators further reinforce the coupled nature of soil biochemical health and plant nutritional value.

Integrating microbial taxonomic patterns with measured enzyme activities offers correlative insights into possible mechanisms underlying metabolic optimization in *C. morifolium* (Supplementary Figure S4). For example, the enrichment of genera such as *Bacillus* and *Scedosporium* coincided with higher protease and urease activities, suggestive of enhanced nitrogen mineralization (Chen Q. et al., 2025; Chen X. et al., 2025). Simultaneously, Co-occurrence patterns involving *Didymella* and sucrase activity were linked to higher soluble sugar levels, indicating possible functional synergy in carbon mobilization.

Nutrient bioavailability is further enhanced by ALP activity—driven by *uncultured Chloroflexi bacterium* and *Cystobasidium*-which facilitates the release of orthophosphate (Chen et al., 2021; Chen X. et al., 2025). Providing the necessary metabolic fuel (ATP) for crude fat accumulation. Additionally, Bacillus-mediated catalase activity stabilizes the soil redox environment and strengthens microbial defenses against oxidative stress, explaining the increased levels of antioxidant compounds like Vitamin C, flavones, and phenolic acids (e.g., 4-HBA and vanillin) (Chen et al., 2019; Tian et al., 2022). These correlations point to a coordinated response across soil chemistry, microbial function, and plant metabolism. They support the idea of a soil-microbe-plant continuum, in which fungal network properties may act as integrative indicators of system health, though direct evidence of causal linkage remains to be established. The transition to integrated management strategies recruits and empowers beneficial fungal lineages that act as essential connectors (high betweenness centrality).

From a practical standpoint, these findings imply that management strategies aiming to bolster fungal positive cohesion, through targeted organic matter quality, residue management, and rotation selection, can simultaneously raise yield and phytochemical quality in *Chrysanthemum morifolium*. However, it is important to note trade-offs: some organic-enriched treatments reduced bacterial functional redundancy and network robustness, potentially increasing vulnerability to perturbations; thus, agronomic designs should balance fungal promotion with maintenance of bacterial functional insurance to ensure long-term system resilience. Future work that manipulates fungal network properties experimentally and tracks fluxes of key nutrients and phenylpropanoid precursors will be valuable to causally link fungal dynamics to the metabolic trajectories documented here. Moreover, the above interpretations draw on our field results and supporting thesis-level literature but would benefit from targeted manipulative experiments (e.g., fungal suppression/enrichment, isotope tracing) and metatranscriptomic or metabolomic analyses to resolve causality and the biochemical pathways connecting fungal activity to phytochemical biosynthesis.

### 4.4 Management implications and limitations

In summary, this study identifies fungal positive cohesion as a robust correlate of enhanced yield and quality in *Chrysanthemum morifolium* under integrated management. These findings generate multiple testable hypotheses regarding the functional importance of fungal interactomes in sustainable agriculture. From a management perspective, our results suggest that integrated rotation with organic amendments, particularly radish-based cropping combined with vermicompost or bio-organic fertilizer, which can effectively alleviate continuous cropping obstacles. These practices consistently improved soil fertility, suppressed predicted pathogens, and promoted cooperative fungal interactions. However, trade-offs exist: some organic-enriched treatments reduced bacterial functional redundancy, potentially compromising long-term resilience. Thus, optimal designs must balance fungal promotion with maintenance of broader microbial insurance.

It is essential to emphasize that the evidence remains associative. The experimental design does not allow us to determine causal direction: while we hypothesize that stable fungal networks promote plant performance through improved nutrient cycling and pathogen suppression, an alternative explanation—reverse causality—is equally plausible. High-performing plants may actively shape their rhizosphere microbiome via increased root exudation or selective recruitment of beneficial fungi, thereby enhancing network cohesion rather than being driven by it. Moreover, unmeasured environmental heterogeneity, historical land use effects, or stochastic assembly processes could act as potential confounding factors. Therefore, observed patterns should be interpreted as ecologically meaningful associations that generate testable hypotheses—not as proven mechanisms.

From a methodological perspective, it should be noted that functional predictions derived from 16S rRNA and ITS amplicon sequencing are *in silico* inferences based on taxonomic proxies and reference databases. They do not reflect actual gene expression, protein activity, or true metabolic function. Therefore, all functional interpretations presented here are considered tentative and hypothesis-generating, pending validation through metagenomic, transcriptomic, or targeted biochemical assays. To translate these ecological insights into practical agricultural strategies, future research should leverage higher-resolution technologies, such as shotgun metagenomics, metatranscriptomics, or single-cell genomics, to move beyond taxonomic inference and directly characterize the functional potential and activity of soil microbial communities. Integrating such advanced approaches will be critical for identifying robust, management-responsive microbial indicators and developing precision microbiome-based interventions that enhance crop productivity and soil health in a sustainable manner.

## 5 Conclusion

This study demonstrates that transitioning from continuous monoculture to integrated rotation systems combined with organic amendments leads to significant changes in soil physicochemical properties, microbial community composition, and crop performance in *Chrysanthemum morifolium*. Among the tested regimes, radish-based rotation and its combination with vermicompost or bio-organic fertilizer emerged as particularly effective strategies for enhancing soil fertility, as evidenced by improved nutrient availability, increased soil organic matter, and elevated enzyme activities associated with carbon and nitrogen cycling. Specifically, organic inputs and diversified residues promote the assembly of a stable, cohesive fungal interactome characterized by enriched Ascomycota and Mortierellomycota, reduced pathogen abundance, and enhanced network connectivity. This restructured fungal community appears to mediate improvements in yield and phytochemical quality through at least three interconnected pathways: (i) intensification of carbon and nitrogen cycling via stimulation of sucrase and protease activities; (ii) suppression of soil-borne diseases through functional displacement of plant pathogens; and (iii) facilitation of resource allocation toward secondary metabolism by stabilizing the rhizosphere environment.

Overall, the results suggest that optimizing soil nutrient status and fostering stable fungal networks are promising strategies for improving chrysanthemum yield and quality. These findings provide a hypothesis-generating framework for future research into ecological intensification in medicinal plant cultivation.

## Data Availability

The data presented in the study are deposited in the National Genomics Data Center (NGDC) repository, accession number PRJCA069066.

## Footnotes

1
https://ngdc.cncb.ac.cn/
