# Supplementary material for: Fungal-mediated enhancement of yield and quality in Chrysanthemum morifolium under integrated rotation and organic management
Source: Front Microbiol. 2026 Sep 10;17:1916214. doi: 10.3389/fmicb.2026.1916214 (PMC13601346; doi:10.3389/fmicb.2026.1916214)
Supplement: Supplementary file 1 [file Table_1.DOCX]

*Supporting Information fo*r

**Fungal-mediated enhancement of yield and quality in *Chrysanthemum morifolium* under integrated rotation and organic management**

This file includes:

Page S1 to S9

Tables S1 to S3

Figures S1 to S4

**Supporting tables**

**Table S1.** One-way ANOVA results for soil property under different treatments (mean ± SD).

| Treatment | CK | L-C1 | L-C2 | L-C3 | M-B1 | M-B2 | M-B3 | Y-A1 | Y-A2 | Y-A3 |
| --- | --- | --- | --- | --- | --- | --- | --- | --- | --- | --- |
| AN  (mg kg^-1^) | 150.51±1.47  abc | 208.09±8.68  d | 133.81±2.31  ab | 125.04±9.67  a | 209.13±0.56  d | 208.80±1.66  d | 179.35±0.23  bcd | 151.62±11.14  abc | 188.89±9.55  cd | 160.25±  66.89abcd |
| AP  (mg kg^-1^) | 4.78±0.56  e | 5.00±0.09  e | 7.46±0.14f | 4.19±0.10  d | 2.28±0.05  b | 2.46±0.29  b | 1.63±0.06  a | 2.54±0.05  b | 2.12±0.08  b | 3.25±0.10  c |
| pH | 7.73±0.11  cd | 7.68±0.03  abc | 7.67±0.06  abc | 7.71±0.04b  cd | 7.80±0.03  d | 7.58±0.01  a | 7.68±0.04  abc | 7.73±0.02  cd | 7.61±0.01  ab | 7.66±0.03  abc |
| AK  (mg kg^-1^) | 19.14±0.68  c | 17.59±0.24  b | 25.87±0.17  e | 28.05±0.41  f | 18.90±0.02  c | 22.94±0.98  d | 33.67±0.11  g | 13.26±0.13  a | 27.97±0.87  f | 12.81±0.31  a |
| SOM  (%) | 3.07±0.28 | 3.51±0.10 | 3.62±0.11 | 3.45±0.00 | 3.07±0.48 | 3.53±0.15 | 2.93±0.02 | 3.42±0.19 | 3.29±0.24 | 3.30±0.13 |
| ORP  (mV) | -41.30±0.71  c | -41.75±0.64  c | -44.60±0.99  b | -39.05±0.78  d | -43.80±0.42  b | -46.35±0.64  a | -26.30±0.71  f | -37.20±0.42  e | -24.60±0.42  g | -12.45±0.21  h |

Different superscripts represent statistical significance between different types (*p* < 0.05). AN: soil available nitrogen; AP: soil available phosphorus; AK: soil available potassium; SOM: soil organic matter; ORP: soil oxidation-reduction potential;

**Table S2.** One-way ANOVA results for yield traits of *C. morifolium* under different treatments (mean ± SD).

|  | CK-1 | L-C1 | L-C2 | L-C3 | M-B1 | M-B2 | M-B3 | Y-A1 | Y-A2 | Y-A3 |
| --- | --- | --- | --- | --- | --- | --- | --- | --- | --- | --- |
| Disc Floret Count | 14.0±2.7  c | 14.0±1.0c | 7.0±1.0  a | 8.0±2.0  ab | 13.7±2.5  c | 7.7±2.9  a | 9.3±4.0  abc | 13.0±1.0  bc | 7.3±1.2  a | 14.0±7.0  c |
| Inflorescence Diameter | 9.2±1.0 | 10.4±0.4 | 11.5±0.5 | 10.2±1.0 | 10.0±0.0 | 10.2±1.2 | 9.8±1.4 | 10.0±0.5 | 10.8±1.0 | 9.9±0.8 |
| Fresh Weight per Plant | 187.1±30.6 | 189.1±65.5 | 232.6±101.0 | 188.5±55.8 | 188.2±17.5 | 286.8±73.6 | 222.1±59.9 | 317.1±99.7 | 338.7±105.2 | 258.1±70.0 |
| Ray Floret Count | 268.3±28.3  a | 296.3±15.5ab | 344.7±20.1  de | 316.0±10.6bcd | 300.0±38.6abc | 339.7±38.9  cde | 324.0±11.3  bcd | 274.3±22.2  a | 365.3±14.6  e | 347.7±19.6de |
| Plant Height | 33.2±4.3  a | 35.3±3.5  ab | 43.9±1.6  c | 43.0±2.4  c | 41.4±3.6  bc | 45.3±3.9  cd | 45.3±1.8  cd | 45.6±3.2  cd | 55.8±9.0  e | 52.2±2.8  de |
| Crown Spread | 32.4±1.6  abc | 28.8±4.9  ab | 32.6±2.4  abc | 26.3±2.9  a | 37.0±9.9  bcd | 40.4±5.4  cd | 39.4±6.0  cd | 43.3±5.3  d | 41.9±2.2  cd | 45.3±8.9  d |
| Flowers per Plant | 37.0±6.1  a | 36.0±12.5  a | 55.3±27.4  abc | 43.0±12.8  ab | 43.0±4.0  ab | 74.0±19.0  bc | 55.3±15.0  abc | 85.3±27.0  c | 87.0±27.1  c | 69.3±18.9  bc |

Different superscripts represent statistical significance between different types (*p* < 0.05).

**Table S3.** One-way ANOVA results for plant nutrients and secondary metabolites under different treatments (mean ± SD).

| Treatment | CK | L-C1 | L-C2 | L-C3 | M-B1 | M-B2 | M-B3 | Y-A1 | Y-A2 | Y-A3 |
| --- | --- | --- | --- | --- | --- | --- | --- | --- | --- | --- |
| CF | 0.01±0.00  a | 0.02±0.00  d | 0.03±0.00  e | 0.02±0.00  d | 0.03±0.00  e | 0.02±0.00  c | 0.02±0.00  c | 0.01±0.00  b | 0.01±0.00  a | 0.01±0.00  b |
| SS | 26.13±0.92  a | 71.85±1.81  f | 38.94±3.88  b | 50.25±0.47  cd | 53.49±2.23  d | 65.66±3.36  e | 65.97±3.75  e | 47.19±1.09  c | 36.13±0.86  b | 45.83±1.01  c |
| SP | 25.52±1.13  g | 16.43±0.02  f | 11.41±0.21  d | 6.84±0.00  b | 14.43±0.51  e | 5.18±0.05  a | 15.54±0.10  f | 5.19±0.01  a | 7.85±0.03  c | 5.54±0.12  a |
| VC | 0.88±0.01  e | 0.89±0.01  e | 0.87±0.03  e | 0.81±0.03  d | 0.54±0.01  a | 0.64±0.01  b | 0.66±0.05  b | 0.71±0.02  c | 1.00±0.00  f | 0.65±0.02  b |
| AA | 3.68±0.08  f | 2.43±0.15  e | 2.12±0.18  cd | 2.31±0.02  de | 1.84±0.02  bc | 1.71±0.12  ab | 2.00±0.08  bc | 1.86±0.28  bc | 2.09±0.15  cd | 1.44±0.08  a |
| Flav | 8.76±0.27 | 8.96±0.34 | 8.74±0.47 | 8.72±0.02 | 8.70±0.02 | 8.57±0.16 | 8.88±0.02 | 8.97±0.31 | 8.72±0.02 | 8.77±0.24 |
| 4-HBA | 0.24±0.01  a | 0.55±0.02  d | 0.93±0.02  f | 1.21±0.01  g | 0.34±0.01  b | 0.21±0.01  a | 0.35±0.04  bc | 0.56±0.03  d | 0.39±0.01  c | 0.69±0.00  e |
| VA | 1.70±0.08  bc | 1.40±0.01  a | 2.06±0.17  d | 2.40±0.00  e | 1.54±0.05  ab | 2.03±0.13  d | 1.54±0.06  ab | 1.76±0.01  c | 1.49±0.10  a | 2.06±0.01  d |
| SA | 0.66±0.03  de | 0.96±0.01  f | 0.56±0.01  cd | 2.48±0.18  g | 0.26±0.01  a | 0.73±0.02  e | 0.47±0.02  bc | 0.48±0.02  bc | 0.37±0.01  ab | 0.58±0.09  cde |
| FA | 0.21±0.02  b | 0.19±0.01  b | 0.29±0.03  cd | 0.55±0.05  f | 0.27±0.02  c | 0.36±0.04  e | 0.21±0.01  b | 0.05±0.00  a | 0.25±0.01  bc | 0.34±0.04  de |

Different superscripts represent statistical significance between different types (*p* < 0.05). CF: Crude Fat; SS: Soluble Sugar; SP: Soluble Protein; VC: Vitamin C; AA: Amino acids; Flav: Flavones;4-HBA: 4-Hydroxybenzoic acid; VA: Vanillin; SA: Succinic acid; FA: Fumaric acid.

**Supporting figure captions**

**Figure S1|** Ternary plots showing the phylum-level community composition across treatments for (a) bacteria and (b) fungi. Each point represents a phylum, with point color indicating taxonomic affiliation and point size scaled to relative abundance. Axes correspond to the proportional contribution of each treatment group in the ternary space.

**Figure S2|** Comparison of soil bacterial functional redundancy index (FRI) across the CK, Rot, Rot_Org and Rot_Org_Ver treatments.

**Figure S3|** SHAP dependence plot grid summarizing the marginal effects of fungal positive cohension (Fun_Pos_Cohesion) on normalized yield of *C. morifolium*. Points show individual observations, the solid curve denotes the fitted trend, and the shaded band indicates the 95% confidence interval.

**Figure S4|** Heatmap of the correlations coupling microbial taxonomic distribution (a, b) with soil enzyme activities and metabolic profiles.

Figure S1

Figure S2

Figure S3

Figure S4
